# High Interleukin-12 Levels May Prevent an Increase in the Amount of Fungi in the Gastrointestinal Tract during the First Years of Diabetes Mellitus Type 1

**DOI:** 10.1155/2016/4685976

**Published:** 2016-12-29

**Authors:** Beata Kowalewska, Katarzyna Zorena, Małgorzata Szmigiero-Kawko, Piotr Wąż, Małgorzata Myśliwiec

**Affiliations:** ^1^Department of Tropical Medicine and Epidemiology, Institute of Maritime and Tropical Medicine, Medical University of Gdańsk, Gdańsk, Poland; ^2^Department of Immunobiology and Environment Microbiology, Medical University of Gdańsk, Gdańsk, Poland; ^3^Clinics of Paediatrics, Diabetology and Endocrinology, Medical University of Gdańsk, Gdańsk, Poland; ^4^Department of Nuclear Medicine, Medical University of Gdańsk, Gdańsk, Poland

## Abstract

The objective of the research was to investigate serum levels of interleukin-12 (IL12) in relation to percentage of yeast-like fungi colonies residing in the gastrointestinal tract in children and adolescents with type 1 diabetes mellitus (T1DM). The study involved 83 children and adolescents, including 53 T1DM patients and 30 healthy control subjects. In the studied population biochemical tests were performed and yeast-like fungi were identified in the faeces. Moreover, IL12 absorbance was measured and measurements of* Candida albicans* IgG and IgM antibodies were performed with microplate reader ChroMate 4300 (Awareness Technology, Inc., USA) at wavelength *λ* = 450 nm. In the group of T1DM children and adolescents with disease duration ≤ 2 years, high levels of IL12 were found with lower percentage of yeast-like fungal colonies versus T1DM patients with disease duration > 2 years and ≤5 years, as well as versus T1DM patients with disease duration > 5 years. Additionally, serum levels of IL12 were found to be decreasing by 18.1 pg/ml with each year of diabetes duration. IL12 serum levels were also found to be decreasing by 52.9 pg/ml with each 1% increase in HbA1c. We suggest that high IL12 levels can inhibit infection with yeast-like fungi colonizing the gastrointestinal tract in children and adolescents with T1DM. Further studies are needed to confirm the antifungal activity of IL12.

## 1. Introduction

*Candida albicans* is currently the most common fungal pathogen found in humans and the most common cause of superficial fungal infections of the gastrointestinal mucosa as well as deep organ, systemic infections [1–3]. There are approximately 150 species within the Candida genus, but only 9 of them are considered human pathogens. Recently in clinical practice other Candida species have been detected in addition to* Candida albicans, *which are resistant to many of the currently available antifungal treatments. These include* C. glabrata*,* C. tropicalis*,* C. parapsilosis,* and* C. krusei* [1, 4]. Our previous studies have shown that yeast-like fungi isolated from faecal samples of T1DM children presented with greater species diversity versus those found in control samples. Moreover, more* Candida albicans* strains in this group showed lower susceptibility to the tested drugs and higher enzymatic activity [5].

In physiological conditions* Candida albicans* is a harmless commensal of the gastrointestinal system and does not cause candidiasis [3, 6, 7]. However, in recent years studies have been showing that a number of biochemical and environmental factors related to diseases such as tumors or diabetes can predispose to the development of candidiasis [3, 8, 9]. Furthermore, authors have found considerable differences in the composition of the gut microbiota between diabetic patients and healthy subjects [10–12]. In the mucous membrane of the digestive tract there are various immune cells including numerous T and B lymphocytes, mast cells, granulocytes, and macrophages that are able to trigger immune response. Key immune surveillance cells in the mucosa that recognise and respond to* Candida albicans* are macrophages that exhibit phenotypic plasticity [1, 4, 13]. M1 macrophages may control* Candida* infection through phagocytosis and also induction of inflammation by producing inflammatory cytokines. On the other hand, M2 macrophages may also kill* Candida* by phagocytosis and produce anti-inflammatory cytokines and growth factors leading to immune tolerance to* Candida *[14, 15]. *β*-Glucan is a major cell wall component of* Candida albicans* that binds to Toll-like receptor (TLR) 2 and Dectin-1 receptors on M1 macrophages to trigger downstream cell signalling for tumor necrosis factor-alpha (TNF*α*) production [16, 17]. Recently Kashem et al. reported that the yeast form of* Candida albicans* promotes the development of a protective Th17 response through Dectin-1. Filamentous hyphae, independently of Dectin-1, induce a Th1 response that is protective in a subsequent systemic challenge [18]. In other studies it was observed that* Candida albicans* stimulates TLR2 receptors, triggers immunosuppression, increases interleukin-10 (IL10) synthesis, and increases TregCD4^+^CD25^+^ cell survival rate [6]. It has been well documented that IL10 plays an inhibitory role in monocytes and neutrophils against Candida [19]. In the murine models of candidiasis, neutralization of IL10 upregulates nitric oxide production and protects susceptible mice from challenge with* Candida albicans*. Studies in patients with T2DM showed that PBMCs from pathogens stimulated with Candida albicans are characterized by significantly lower interferon-gamma (IFN*γ*) production. However, there were no significant differences detected in respect of levels TNF*α* and interleukin-6 (IL6) [20].

Interleukin-12 (IL12) is an important immunoregulatory cytokine that is produced mainly by antigen-presenting cells (APCs). IL12 has multiple biological functions. It is produced mainly by phagocytes (monocytes/MΦ and neutrophils) and dendritic cells, in response to pathogens (bacteria, viruses, intracellular parasites, and yeast-like fungi) [21–23]. The role of IL12 in defence mechanisms protecting against* Candida albicans* infections has been presented in experimental murine model [24–26]. IL12 deletion leads to acute susceptibility to oral infection with yeast* Candida albicans*, whereas such mice are resistant to systemic disease [25]. No data are available on the IL12 status in the context of yeast-like fungal infections in T1DM children and adolescents. Therefore, the objective of our research was to investigate the relationship between IL12 levels in serum and yeast-like fungal colonies in the digestive tract of children and adolescents with T1DM.

## 2. Patients and Methods

The study involved 53 adolescent patients (19 girls and 34 boys) with T1DM. All patients were under intensive insulin therapy (0.9 ± 0.2 IU of insulin per day/kg of body weight). Diabetes was diagnosed according to the Polish Diabetes Association guidelines which correspond with the guidelines of the WHO [27, 28]. Glycated hemoglobin (HbA1c) was measured with an immunoturbidimetric method using a Unimate 3 set (Hoffmann-La Roche AG, Basel, Switzerland). Moreover, in the studied population of T1DM patients no long-term systemic complications were seen such as retinopathy, nephropathy, or neuropathy. Children and adolescents had not been receiving antibiotics for up to 3 months prior to participation in the study. Children and adolescents with symptoms of infection or systemic somatic illness other than diabetes mellitus were excluded from the study.

Control group consisted of 30 healthy children and adolescents, age and BMI matched (16 girls and 14 boys). Patients with T1DM and their matched controls were examined by a pediatrician on the day of collection of the faecal samples. Medical history was taken and physical examination was performed and did not reveal any gastrointestinal complaints in either study group. Written informed consent was obtained from all children and adolescents participating in the study or from their parent or guardian. The study was approved by the Ethics Committee of the Medical University of Gdańsk (NKBBN/125/2014) and the investigation was carried out in accordance with the principles of the Declaration of Helsinki, as revised in 1996.

### 2.1. Yeast-Like Fungal Cultures

**Figure 1:**
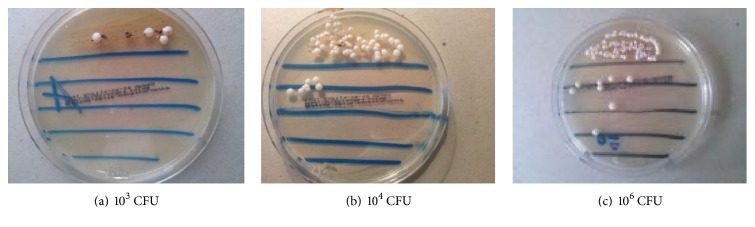


The study materials were samples of fresh faeces collected from the study participants. The samples were collected into sterile containers and provided to the laboratory on the day of collection. On the same day the samples were used to establish cultures. In order to isolate and enumerate yeast-like fungal colonies in 1 g faeces, quantitative cultures on Sabouraud Dextrose Agar were used. Faecal suspensions in normal saline and serial dilutions 1 : 10, 1 : 100, 1 : 1000, and 1 : 10000 were prepared. Five growth lines were drawn on the Sabouraud medium plate. On the first line 10 *μ*g faces were equally spread. Then 10 *μ*g of each faecal dilution was plated and equally spread on each subsequent line, respectively. The cultures were incubated for 72 h at 37°C. Then the number of fungal colonies that had grown on the plate was counted. According to the plate dilution, the number was converted into colony-forming units in 1 g faeces. Growth units were established as 10^3^–10^6^ CFU/g. Example values for colony-forming units in 1 g faeces are represented (Figures 1(a)–1(c)).

### 2.2. Measurements of* C. albicans* IgG and IgM Antibodies

*C. albicans* IgG and IgM antibodies were measured with commercially available ELISA tests by* DRG Instruments GmbH*, D-35039 Marburg, Germany. Absorbance was read at 450 nm on the automated plate reader ChroMate 4300 (Awareness Technology, Inc., USA).

Index values for* C. albicans* IgG and IgM antibody absorbance measurements were calculated according to the following formula and expressed in DRG units (DU), as instructed by the manufacturer:

(1) Patient (mean) absorbance value×10CO=DRG Units=DU.

Index values for* C. albicans* IgG and IgM antibodies < 9 DU were considered negative. The values between 9 and 11 were classified as “gray zone,” informing that the result was inconclusive.* C. albicans* IgG and IgM antibodies > 11 DU indicated positive result.

### 2.3. Detection in Serum of IL12 Levels

Blood samples were immediately placed on ice, clarified by centrifugation at 3.000*g* for 5 min at 4°C, and kept frozen at −80°C until assayed. Serum level of IL12 was measured by immunoenzymatic ELISA method (Quantikine High Sensitivity Human by R&D Systems, Minneapolis, MN, USA) according to manufacturer protocol. Minimum detectable concentrations were determined by the manufacturer as 1.0 pg/ml. Intra-assay was 1.1% and interassay 7.1%. Precisions performances of the assays were determined on 20 replicates from the quality control data of the laboratory. Absorbance was read at 450 nm on an automated plate reader ChroMate 4300 (Awareness Technology, Inc., USA). The reference curve was prepared according to the manufacturer's recommendations.

### 2.4. Statistical Analyses

Statistical analyses were performed with the RKWard Data Analysis Tool Version 0.6.1 using the KDE Development Platform 4.13.3 [29]. The Shapiro-Wilk *W* test was used to verify whether the quantitative variable came from normally distributed population. To investigate differences for the two data sets Snedecor's *F*-test for homogeneity of variances was performed. The Wilcoxon Mann–Whitney *U* test was used to confirm equality of means. In case of homogeneity Student's *t*-test was used for two means. For larger data set the analysis of variance was conducted with Bartlett's test (more advanced form of Snedecor's *F*-test for a higher number of samples). The differences between the study groups were tested with Kruskal-Wallis test. For groups with homogenous variance ANOVA test was applied. Moreover, Chi-square test for independence was used for qualitative variables. In order to verify which of the studied clinical and biochemical parameters significantly affects IL12 in T1DM patients, a multivariate and univariate linear regression model was applied. The next step used a Stepwise Algorithm with AIC (Akaike Information Criterion) statistics in order to find the best of independent variables [30]. The significance level was set at 0.05.

## 3. Results

### 3.1. Clinical Characteristics of T1DM Children and Adolescents

The group of children and adolescents with T1DM had significantly higher HbA1c levels (*P* = 0.005), higher C-reactive protein levels (*P* = 0.003), higher IL12 levels (*P* = 0.003), and higher* C. albicans* IgG antibody level (*P* = 0.049) versus control subjects. No* Candida albicans *IgM antibodies were detected, either in the serum of T1DM patients or in the serum of control group. Clinical characteristics of the study groups are shown in Table 1.

### 3.2. The Amounts of Yeast-Like Fungi in Faecal Samples of T1DM Children and Adolescents

The study enrolled 53 children and adolescents with T1DM and 30 healthy control subjects. No yeast-like fungi were detected in 13/53 faecal samples from T1DM patients and in 9/30 faecal samples from control group. In 40 faecal samples from T1DM patients and in 21 faecal samples from control subjects yeast-like fungal growth was detected at the level of 10^3^–10^6^ CFU/g. In T1DM patients, out of 40 faecal samples from which yeast-like fungi were cultured, in 33 (82.5%) cases there was only a single species of yeast-like fungi detected and in 7 (17.5%) patients with T1DM there were two different species detected. In positive faecal samples of children with T1DM there were 47 strains of yeast-like fungi. The cultured yeast-like fungi in positive faecal samples from T1DM children belonged to 9 different species.* Candida* ssp. constituted 82.98% of all isolated strains, and the remaining 17.02% was constituted by other yeast-like fungi. Among all cultured strains, prevalent was Candida albicans, which constituted 61.7% of all the strains cultured. Other species of* Candida* type included* C. krusei* (8.5% of all fungi),* C. famata* (6.38%),* C. parapsilosis (2.12%), C. lusitaniae (2.12%), and C. guilliermondii* (2.12%). Other isolated species of yeast-like fungi included* Rhodotorula* 8.5%,* Geotrichum* 6.38%, and* Saccharomyces* 2.12%.

Among 30 healthy control subjects fungi were detected in faecal samples from 21 (70%) children. In the control group we detected 21 strains of yeast-like fungi. The cultured strains belonged to 4 species. Among all cultured strains, prevalent was* Candida albicans*, which constituted 85.7% of all the yeast-like fungi strains that were cultured. Moreover,* C. famata* and* C. tropicalis *were detected, representing each (4.76%) of all fungi. The last fungal species detected was* Rhodotorula* sp., not being a member of the Candida genus, which accounted for 4.76% of isolates.

### 3.3. Clinical Characteristics of T1DM Children and Adolescents according to Their Disease Duration

The subgroup of children and adolescents with T1DM duration ≤ 2 years was significantly younger versus T1DM patients with their diabetes duration > 2 years and ≤5 years (*P* = 0.053) and versus T1DM patients with their diabetes duration > 5 years (*P* = 0.000). Moreover, the subgroup of children with T1DM for ≤2 years had significantly lower HbA1c levels versus the subgroup with T1DM duration > 2 and ≤5 years (*P* = 0.02) and versus the subgroup with T1DM duration > 5 years (*P* = 0.01). No statistically significant differences were seen between the study subgroups in hsCRP levels (*P* = 0.23; *P* = 0.74; *P* = 0.41, resp.). In addition, higher IL12 levels were seen in the serum of T1DM children and adolescents with duration of diabetes ≤ 2 years and the amount of fungi 10^3^–10^6^ CFU versus T1DM children and adolescents with duration of diabetes > 2 and ≤5 years and versus T1DM patients with duration of diabetes > 5 years. No significant differences were seen in IL12 levels in patients with duration of diabetes > 2 and ≤5 years versus patients with duration of diabetes > 5 years (*P* = 0.70) (Table 2).

### 3.4. Serum IL12 Levels in Children and Adolescents in the Course of T1DM

**Figure 2:**
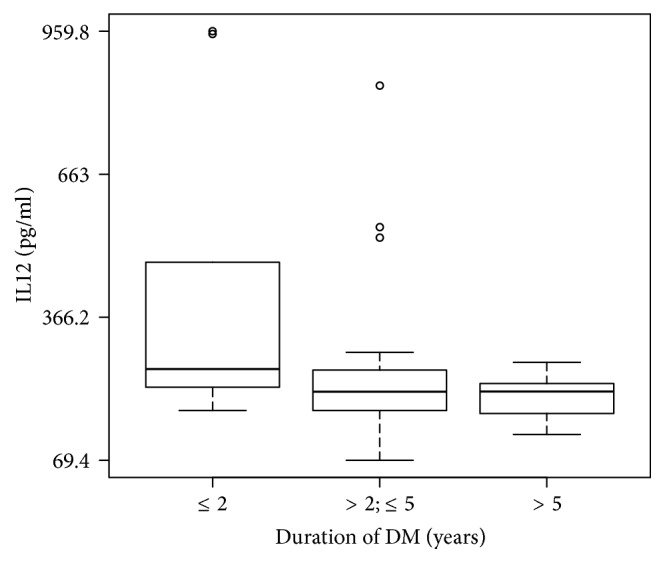
Serum IL12 levels in children and adolescents in the course of T1DM.

In the studied population higher IL12 levels were seen in the serum of T1DM children and adolescents with duration of diabetes ≤ 2 years and the amount of fungi 10^6^ versus T1DM children and adolescents with duration of diabetes > 2 and ≤5 years (^*∗*^*P* = 0.04), and versus T1DM patients with duration of diabetes > 5 years (^*∗∗*^*P* = 0.008). No significant differences were seen in IL12 levels in patients with duration of diabetes > 2 and ≤5 years versus patients with duration of diabetes > 5 years (*P* = 0.70) (Figure 2).

### 3.5. Measurements of* Candida albicans* IgG and IgM Antibodies in the Serum of T1DM Children and Adolescents

No* Candida albicans* IgG antibodies were detected in the subgroup of children from diabetes for ≤2 years. In the subgroup of patients with history of diabetes lasting between >2 and ≤5 years the level of* Candida albicans* IgG antibodies was higher versus the subgroup with duration of diabetes > 5 years, although the difference was not statistically significant (*P* = 0.45). The* Candida albicans* IgM antibodies were not detected, either in the serum of T1DM patients or in serum of control group (Table 3).

### 3.6. Analysis of Variance (ANOVA) for Double Classification

In the next step of the research an analysis of variance (ANOVA) for double classification has shown that duration of diabetes and yeast-like fungal colonies in the amount of 10^3^–10^6^ CFU/g significantly affect IL12 levels (^*∗*^*P* = 0.01) (Table 4).

In the studied population of T1DM children and adolescents a statistically significant correlation was seen between HbA1c levels and duration of diabetes and the amount of yeast-like fungi (^*∗*^*P* = 0.05) (Table 5).

In the studied population of T1DM children and adolescents no statistically significant effect of duration of diabetes and yeast-like fungal colonies on hsCRP levels was seen (*P* = 0.42) (Table 6).

### 3.7. Multivariate Regression

In order to verify which of the studied clinical and biochemical parameters significantly affects IL12 in T1DM patients, a multivariate and univariate linear regression model was applied (Tables 7–10). Initially there were four parameters included in the multivariate regression model: disease duration, HbA1c, hsCRP, and the number of yeast-like fungi colonies detected in T1DM patients.

The next step used a Stepwise Algorithm with AIC (Akaike Information Criterion) statistics in order to find the best of independent variables.

Minimization of the AIC statistics allowed us to select independent variables of HbA1c and diabetes duration as optimal ones for multiple linear regression model.

### 3.8. Univariate Linear Regression

A univariate linear regression model was used to investigate, which of the studied clinical and biochemical parameters could affect IL12 levels in patients with T1DM (Tables 9-10). In the studied group of T1DM children and adolescents serum levels of IL12 decreased by 18.1 pg/ml with each year of diabetes duration, *β* = −18.1, *P* < 0.05. Serum IL12 levels also decreased by 52.9 pg/ml with each 1% increase in HbA1c levels; *β* = −52.9 *P* < 0.05.

Univariate regression showed that in T1DM children with an increase of HbA1c by 1%, the level of IL12 in serum decreases by 52.9 pg/ml (*P* < 0.05).

### 3.9. Univariate Correlation between Serum IL12 Levels and Duration of Diabetes and HbA1c in T1DM Patients

**Figure 3:**
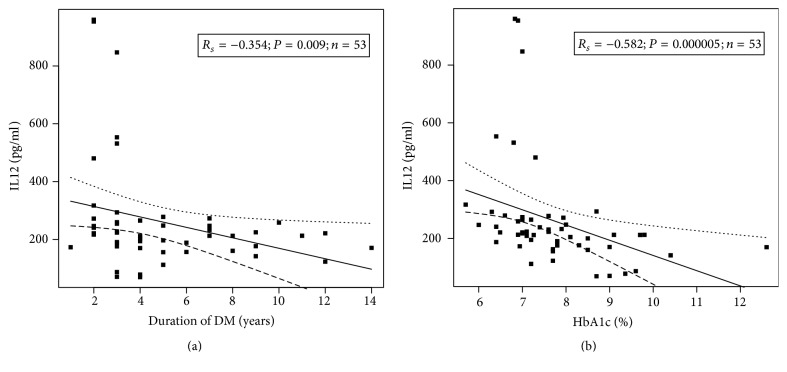
The negative correlation between IL12 levels and duration of diabetes, *R*_*s*_ = −0.354 and *P* = 0.009 (a), as well as between IL12 and HbA1c levels, *R*_*s*_ = −0.582 and *P* = 0.000005 (b).

In T1DM patients, we observed a statistically significant negative correlation between IL12 levels and duration of diabetes,* R*_*s*_ = −0.354, *P* = 0.009 (Figure 3(a)), as well as between IL12 and HbA1c levels,* R*_*s*_ = −0.582, *P* = 0.000005 (Figure 3(b)). There were no significant correlations between other baseline variables.

## 4. Discussion

In our study we observed higher level, although with normal range of* Candida albicans* IgG antibodies in serum of T1DM patients, compared to the control group. Detection of* Candida albicans* IgG antibodies indicates past infection. The result of a single antibody test, interpreted without other mycological tests, imaging diagnostics, and evaluation of the patient's clinical condition, does not allow for differentiation between colonization, local forms of candidiasis, and the invasive form of infection [31–33]. In our study positive higher level of* Candida albicans* IgG antibodies most probably results from reaction to the contact of yeast-like fungi cells with elements of serum of T1DM patients. Due to increased intestinal permeability in T1DM patients there is increased risk of transfer of the fungus antigen to the vascular bed [34, 35]. In our group of patients with T1DM the level of* Candida albicans* IgM antibodies in serum was negative, which indicates lack of ongoing gastrointestinal infection in children and teenagers with T1DM. What is more, during the study, children and adolescents with T1DM did not report any gastrointestinal complaints.

The key finding of our research is that children and adolescents at early stages of diabetes, up to 2 years' duration, have been found to present with low percentage of yeast-like fungal colonies but with high IL12 level versus patients with duration of diabetes > 2 years and ≤5 years, as well as versus patients with duration of diabetes > 5 years. Analysis for double classification has shown that duration of diabetes and fungal colonies in the amount of 10^3^–10^6^ CFU/g significantly affect IL12 levels. Moreover, linear regression analysis has shown that serum levels of IL12 decreased by 18.1 pg/ml with each year of diabetes duration.

The role of IL12 in the course of diabetes and chronic complications is enigmatic. Twenty years ago, in 1996, Rothe et al. in their studies of nonobese diabetic (NOD) mice demonstrated that IL12 contributed to the development of T1DM [36]. In the same year potent antiangiogenic properties of IL12 were discovered [37]. Therefore, at the same time a duality of IL12 was proven [36, 37]. In view of these findings there is no doubt that T1DM show dominant Th1 cytokine profile with overexpression of IL12, which contributes to the “outbreak” of type 1 diabetes mellitus [25, 38]. However with continued diabetes, IL12 acts as antiangiogenic factor, which has been shown in our previous studies and studies by other authors [39–42]. We are now suggesting that IL12 may also prevent fungal infections. In our opinion these are the first evidences to prove the protective role of IL12 against yeast-like fungal infections in the gastrointestinal tract of children and adolescents with T1DM. Currently available experimental studies confirm our suggestions. Studies of* C. albicans*-infected mice have revealed that IL12p40 knockout mice showed considerably higher* C. albicans* colonization versus mice with high IL12 levels [26]. Moreover, IL12p40 knockout mice were more susceptible to gastrointestinal candidiasis rather than to systemic infections [26, 27].

Although increased IL12 levels have been found to correlate with severity of inflammation in the course of diabetes, in the early stages of diabetes IL12 may inhibit expression of inflammation as a negative feedback rather than promote inflammatory state [43–45]. In our study no correlation has been found between serum levels of hsCRP and IL12. Moreover, there was no significant difference between T1DM children and adolescents with duration of diabetes ≤ 2 years and the amount of fungal colonies 10^3^–10^6^ CFU/g versus T1DM children and adolescents with duration of diabetes > 2 and ≤5 years and versus T1DM patients with duration of diabetes > 5 years. This lack of correlation between hsCRP and IL12 levels can be explained by the fact that inflammation and resultant immune response are most noticeable during the first months of the disease due to the pancreatic islet *β* cell destruction [46]. During the next few years of their diabetes children and adolescents were seen to present with mild inflammatory state, but when duration of diabetes was longer than 5 years chronic inflammatory state with detectable markers developed [40, 47]. Studies by other authors did not reveal any difference in hsCRP levels in patients with duration of diabetes up to 1 year versus T1DM patients with history of diabetes longer than 1 year either [47, 48]. On the other hand, lack of difference in hsCRP levels and lack of correlation with serum IL12 levels may suggest “good metabolic memory” and persistence of stable inflammatory state [49, 50]. Chronic hyperglycaemia is accompanied by inflammatory state in patients with longer duration of type 1 diabetes [47, 48]. In our study the subgroup of children and adolescents with the shortest duration of diabetes, up to 2 years, showed good metabolic control with HbA1c levels 6.9 ± 0.6%. Mean HbA1c levels were increasing along with duration of diabetes and reached its highest value 8.1 ± 1.3 in patients with duration of diabetes more than 5 years. Finally, the analysis of variance has shown that serum IL12 levels, along with duration of diabetes, are correlated with prevalence of yeast-like fungi in patients with T1DM. The results of our research show that in T1DM patients with up to 5-year long duration of diabetes, high IL12 levels, and good metabolic control the prevalence of yeast-like fungi in the digestive tract is maintained of normal range. So, IL12 overproduction during the first years of diabetes probably not only helps maintain status-quo with no angiopathic complications [39, 40] but also can protect against infections with yeast-like fungi colonizing the digestive tract of children with T1DM. We suggest that high IL12 levels may prevent yeast-like fungal prevalence in the digestive tract of T1DM patients with short duration of diabetes and good metabolic control.

## Figures and Tables

**Table 1:** Clinical characteristics of T1DM children and adolescents versus control subjects.

	T1DM children and adolescents	Control subjects	*P* value
Age (years)	10.9 ± 3.9	10.3 ± 4.9	*P* = 0.588

Duration of diabetes (years)	5.0 ± 3.0	—	—
HbA1c (%)	7.72 ± 1.24	4.3 ± 0.87	*P* = 0.005^**∗**^

hsCRP (mg/L)	2.45 ± 2.27	0.585 ± 0.36	*P* = 0.003^**∗**^

IL12 (pg/ml)	261.1 ± 187.8	50.12 ± 26.53	*P* = 0.003^**∗**^

Cand IgG (DU)	15.8 ± 17.7	4.60 ± 3.08	*P* = 0.049^**∗**^

Cand IgM (DU)	Not detected (—)	Not detected (—)	—

Data are presented as means ± s.d. ^*∗*^Group with T1DM children and adolescents versus control subjects.

**Table 2:** Clinical characteristics of T1DM children and adolescents according to their disease duration.

	Duration of diabetes			*P* value
	Duration of diabetes ≤ 2 years	Duration of diabetes > 2; ≤5 years	Duration of diabetes > 5 years	
Age (years)	8 ± 2.7	10.4 ± 4.0	13.5 ± 2.5	*P* = 0.053^*∗*^ *P* = 0.000^*∗∗*^ *P* = 0.003^*∗∗∗*^

HbA1c%	6.9 ± 0.6	7.8 ± 1.3	8.1 ± 1.3	*P* = 0.02^*∗*^ *P* = 0.01^*∗∗*^ *P* = 0.36

hsCRP mg/dl	2.7 ± 2.3	2.0 ± 1.9	3.0 ± 2.8	*P* = 0.23 *P* = 0.74 *P* = 0.41

IL12 pg/ml	408 ± 301	242 ± 165	201.5 ± 42.5	*P* = 0.04^*∗*^ *P* = 0.008^*∗∗*^ *P* = 0.70

Yeast-like fungi 10^3^–10^6^ CFU/g	21%	37%	42%	*P* = 0.54

Data are presented as means ± SD. ^*∗*^Group with T1DM and duration of diabetes ≤ 2 years versus group > 2; ≤5 years. ^*∗∗*^Group with T1DM and duration of diabetes ≤ 2 years versus group > 5 years. ^*∗∗∗*^Group with T1DM and duration of diabetes > 2; ≤5 years versus group > 5 years.

**Table 3:** Measurements of *Candida albicans* IgG and IgM antibodies in the serum of T1DM children and adolescents.

	Duration of diabetes			*P* value
	Duration of diabetes ≤ 2 years	Duration of diabetes > 2; ≤5 years	Duration of diabetes > 5 years	
*Candida albicans* IgG (DU)	Not detected (—)	19.4 ± 20.2 (+)	14.9 ± 15.3 (+)	*P* = 0.45
*Candia albicans* IgM (DU)	Not detected (—)	Not detected (—)	Not detected (—)	—

**Table 4:** Assessment of the impact of diabetes duration and yeast-like fungal colonies on IL12 levels.

DV: IL12	*β*	SD	*F*-value	*P*(>*F*)
Duration of diabetes	282559	141280	4.4738	0.01^*∗*^
Yeast-like fungi 10^3^–10^6^ CFU/g	3185	3185	0.1009	0.75

A statistically significant correlation was seen between IL12 levels and duration of diabetes and the amount of yeast-like fungi (^*∗*^*P* = 0.01). ANOVA for double classification.

**Table 5:** Analysis of variance (ANOVA) for double classification: effect of diabetes duration and yeast-like fungal colonies on HbA1c level.

DV: HbA1c	*β*	SD	*F*-value	*P*(>*F*)
Duration of diabetes	8.950	4.4749	3.1152	0.05^*∗*^
Yeast-like fungi 10^3^–10^6^ CFU/g	1.123	1.1232	0.7819	0.381

A statistically significant correlation was seen between HbA1c levels and duration of diabetes and the amount of yeast-like fungi (^*∗*^*P* = 0.05). ANOVA for double classification.

**Table 6:** Analysis of variance (ANOVA) for double classification: effect of diabetes duration and yeast-like fungal colonies on hsCRP levels.

DV: hsCRP	*β*	SD	*F*-value	*P*(>*F*)
Duration of diabetes	9.232	4.6159	0.8830	0.42
Yeast-like fungi 10^3^–10^6^ CFU/g	2.280	2.2801	0.4362	0.51

The negative correlation was seen between duration of diabetes and yeast-like fungal colonies on hsCRP levels (*P* = 0.42). ANOVA for double classification.

**Table 7:** Results of multiple regression analysis with IL12 as the dependent variable.

Variable	*β*	SD	*t*-value	*P*(>|*t*|)
Intercept	662.78	156.96	4.22	0.0001
hsCRP	1.16	11.33	0.10	0.92
Yeast-like fungi 10^3^–10^6^ CFU/g	6.61	50,90	0.13	0.90
HbA1c	−44.65	21.46	−2.08	0.04
Duration of diabetes	−12.98	8.51	−1.52	0.13
Multiple *R*-squared = 0.16				
Adjusted *R*-squared = 0.09				
AIC = 554.4				

Dependent variable: IL12; independent variable: HbA1c, hsCRP, duration of diabetes, and yeast-like fungi 10^3^–10^6^ CFU/g.

**Table 8:** Results of the stepwise algorithm with the minimum value of the AIC parameter.

Variable	*β*	SD	*t*-value	*P*(>|*t*|)
Intercept	664.17	152.57	4.35	0.00006
HbA1c	−43.90	20.34	−2.16	0.04
Duration of diabetes	−13.06	8.31	−1.57	0.12
Multiple *R*-squared = 0.16				
Adjusted *R*-squared = 0.13				
AIC = 550.42				

Dependent variable: IL12; independent variable: HbA1c and duration of diabetes; AIC (Akaike An Information Criterion).

**Table 9:** Results of univariate regression analysis with IL12 as the dependent variable.

Variable	*β*	SD	*t*-value	*P*(>|*t*|)
Intercept	350.2	47.6	7.3	0.000000001
Duration of diabetes	−18.1	8.3	−2.1	0.03
Multiple *R*-squared = 0.09				
Adjusted *R*-squared = 0.07				

Dependent variable: IL12; independent variable: duration of diabetes, *P* < 0.05.

**Table 10:** Results of univariate regression analysis with IL12 as the dependent variable.

Variable	*β*	SD	*t*-value	*P*(>|*t*|)
Intercept	669.1	154.7	4.3	0.00007
HbA1c	−52.9	19.8	−2.6	0.01
Multiple *R*-squared = 0.12				
Adjusted *R*-squared = 0.10				

Dependent variable: IL12; independent variable: HbA1c, *P* < 0.05.

## Abbreviations

APCs: Antigen-presenting cells
BMI: Body mass index
CRP: C-reactive protein
CFU: Colony-forming unit
DBP: Diastolic blood pressure
DV: Dependent variable
HbA1c: Glycated hemoglobin
IL10: Interleukin-10
IL12: Interleukin-12
IL6: Interleukin-6
IFN*γ*: Interferon-gamma
TNF *α*: Tumor necrosis factor-alpha
T1DM: Diabetes mellitus type 1
T2DM: Diabetes mellitus type 2
TLR 2: Toll-like receptor 2
SBP: Systolic blood pressure.

## References

[1] Arendrup M. C. (2013). Candida and candidaemia: susceptibility and epidemiology. *Danish Medical Journal*.

[2] Hernday A. D., Noble S. M., Mitrovich Q. M., Johnson A. D. (2010). Genetics and molecular biology in *Candida albicans*. *Methods in Enzymology*.

[3] Gow N. A. R., van de Veerdonk F. L., Brown A. J. P., Netea M. G. (2012). *Candida albicans* morphogenesis and host defence: discriminating invasion from colonization. *Nature Reviews Microbiology*.

[4] Dühring S., Germerodt S., Skerka C., Zipfel P. F., Dandekar T., Schuster S. (2015). Host-pathogen interactions between the human innate immune system and Candida albicans-understanding and modeling defense and evasion strategies. *Frontiers in Microbiology*.

[5] Kowalewska B., Zorena K., Szmigiero-Kawko M., Wąż P., Myśliwiec M. (2016). Higher diversity in fungal species discriminates children with type 1 diabetes mellitus from healthy control. *Patient Preference and Adherence*.

[6] Netea M. G., Sutmuller R., Hermann C. (2004). Toll-like receptor 2 suppresses immunity against *Candida albicans* through induction of IL-10 and regulatory T cells. *The Journal of Immunology*.

[7] Mayer F. L., Wilson D., Hube B. (2013). Candida albicans pathogenicity mechanisms. *Virulence*.

[8] Cheng S.-C., Joosten L. A. B., Kullberg B.-J., Netea M. G. (2012). Interplay between *Candida albicans* and the mammalian innate host defense. *Infection and Immunity*.

[9] Jouault T., Sarazin A., Martinez-esparza M., Fradin C., Sendid B., Poulain D. (2009). Host responses to a versatile commensal: PAMPs and PRRs interplay leading to tolerance or infection by* Candida albicans*. *Cellular Microbiology*.

[10] Boerner B. P., Sarvetnick N. E. (2011). Type 1 diabetes: role of intestinal microbiome in humans and mice. *Annals of the New York Academy of Sciences*.

[11] Soyucen E., Gulcan A., Aktuglu-Zeybek A. C., Onal H., Kiykim E., Aydin A. (2014). Differences in the gut microbiota of healthy children and those with type 1 diabetes. *Pediatrics International*.

[12] Murri M., Leiva I., Gomez-Zumaquero J. M. (2013). Gut microbiota in children with type 1 diabetes differs from that in healthy children: a case-control study. *BMC Medicine*.

[13] Giorgio S. (2013). Macrophages: plastic solutions to environmental heterogeneity. *Inflammation Research*.

[14] Reales-Calderón J. A., Aguilera-Montilla N., Corbí Á. L., Molero G., Gil C. (2014). Proteomic characterization of human proinflammatory M1 and anti-inflammatory M2 macrophages and their response to Candida albicans. *Proteomics*.

[15] Zheng X.-F., Hong Y.-X., Feng G.-J. (2013). Lipopolysaccharide-induced M2 to M1 macrophage transformation for IL-12p70 production is blocked by *Candida albicans* mediated up-regulation of EBI3 expression. *PLoS ONE*.

[16] Vecchiarelli A., Puliti M., Torosantucci A., Cassone A., Bistoni F. (1991). In vitro production of tumor necrosis factor by murine splenic macrophages stimulated with mannoprotein constituents of Candida albicans cell wall. *Cellular Immunology*.

[17] Takahara K., Tokieda S., Nagaoka K., Inaba K. (2012). Efficient capture of Candida albicans and zymosan by SIGNR1 augments TLR2-dependent TNF-*α* production. *International Immunology*.

[18] Kashem S. W., Igyártó B. Z., Gerami-Nejad M. (2015). Candida albicans morphology and dendritic cell subsets determine T helper cell differentiation. *Immunity*.

[19] Roilides E., Anastasiou-Katsiardani A., Dimitriadou-Georgiadou A. (1998). Suppressive effects of interleukin-10 on human mononuclear phagocyte function against *Candida albicans* and *Staphylococcus aureus*. *Journal of Infectious Diseases*.

[20] Zilverschoon G. R. C., Tack C. J., Joosten L. A. B., Kullberg B. J., Van Der Meer J. W. M., Netea M. G. (2008). Interleukin-18 resistance in patients with obesity and type 2 diabetes mellitus. *International Journal of Obesity*.

[21] Trinchieri G. (2003). Interleukin-12 and the regulation of innate resistance and adaptive immunity. *Nature Reviews Immunology*.

[22] Adorini L. (2001). Interleukin 12 and autoimmune diabetes. *Nature Genetics*.

[23] Hamza T., Barnett J. B., Li B. (2010). Interleukin 12 a key immunoregulatory cytokine in infection applications. *International Journal of Molecular Sciences*.

[24] Farah C. S., Hu Y., Riminton S., Ashman R. B. (2006). Distinct roles for interleukin-12p40 and tumour necrosis factor in resistance to oral candidiasis defined by gene-targeting. *Oral Microbiology and Immunology*.

[25] Mencacci A., Cenci E., Del Sero G. (1998). IL-10 is required for development of protective Th1 responses in IL-12- deficient mice upon *Candida albicans* infection. *The Journal of Immunology*.

[26] Netea M. G., Vonk A. G., van den Hoven M. (2003). Differential role of IL-18 and IL-12 in the host defense against disseminated *Candida albicans* infection. *European Journal of Immunology*.

[27] Mohan V., Unnikrishnan R. (2016). *Diabetology: Complications of Diabetes*.

[28] WHO (2006). *Definition, Diagnosis and Classification of Diabetes Mellitus and Its Complications. Report of a WHO Consultation. Part 1: Diagnosis and Classification of Diabetes Mellitus*.

[29] Core Team (2013). *A Language and Environment for Statistical Computing*.

[30] Venables W. N., Ripley B. D. (2002). *Modern Applied Statistics with S*.

[31] De Pauw B., Walsh T. J., Donnelly J. P. (2008). Revised definitions of invasive fungal disease from the European Organization for Research and Treatment of Cancer/Invasive Fungal Infections Cooperative Group and the National Institute of Allergy and Infectious Diseases Mycoses Study Group (EORTC/MSG) Consensus Group. *Clinical Infectious Diseases*.

[32] Pappas P. G., Kauffman C. A., Andes D. R. (2016). Clinical practice guideline for the management of candidiasis: 2016 update by the infectious diseases society of America. *Clinical Infectious Diseases*.

[33] Yera H., Sendid B., Francois N., Camus D., Poulain D. (2001). Contribution of serological tests and blood culture to the early diagnosis of systemic candidiasis. *European Journal of Clinical Microbiology and Infectious Diseases*.

[34] Damci T., Nuhoglu I., Devranoglu G., Osar Z., Demir M., Ilkova H. (2003). Increased intestinal permeability as a cause of fluctuating postprandial blood glucose levels in type I diabetic patients. *European Journal of Clinical Investigation*.

[35] Li X., Atkinson M. A. (2015). The role for gut permeability in the pathogenesis of type 1 diabetes—a solid or leaky concept?. *Pediatric Diabetes*.

[36] Rothe H., Burkart V., Faust A., Kolb H. (1996). Interleukin-12 gene expression is associated with rapid development of diabetes mellitus in non-obese diabetic mice. *Diabetologia*.

[37] Angiolillo A. L., Sgadari C., Tosato G. (1996). A role for the interferon-inducible protein 10 in inhibition of angiogenesis by interleukin-12. *Annals of the New York Academy of Sciences*.

[38] Zhang J., Zhu N., Wang Q. (2010). MEKK3 overexpression contributes to the hyperresponsiveness of IL-12-overproducing cells and CD4^+^ T conventional cells in nonobese diabetic mice. *The Journal of Immunology*.

[39] Zorena K., Myśliwska J., Myśliwiec M., Balcerska A., Lipowski P., Raczyńska K. (2008). Interleukin-12 and tumour necrosis factor-*α* equilibrium is a prerequisite for clinical course free from late complications in children with type 1 diabetes mellitus. *Scandinavian Journal of Immunology*.

[40] Zorena K., Myśliwska J., Myśliwiec M., Balcerska A., Lipowski P., Raczyńska K. (2007). Interleukin-12, vascular endothelial growth factor and tumor necrosis factor-alpha in the process of neoangiogenesis of diabetic retinopathy in children. *Klinika Oczna*.

[41] Zorena K., Kula M., Malinowska E., Raczyńska D., Myśliwiec M., Raczyńska K. (2013). Threshold serum concentrations of tumour necrosis factor alpha (TNF*α*) as a potential marker of the presence of microangiopathy in children and adolescents with type 1 diabetes mellitus (T1DM). *Human Immunology*.

[42] Kleinman M. E., Yamada K., Takeda A. (2008). Sequence- and target-independent angiogenesis suppression by siRNA via TLR3. *Nature*.

[43] Aste-Amezaga M., Ma X., Sartori A., Trinchieri G. (1998). Molecular mechanisms of the induction of IL-12 and its inhibition by IL-10. *The Journal of Immunology*.

[44] Segal B. M., Dwyer B. K., Shevach E. M. (1998). An interleukin (IL)-10/IL-12 immunoregulatory circuit controls susceptibility to autoimmune disease. *Journal of Experimental Medicine*.

[45] Flores R. R., Kim E., Zhou L. (2015). IL-Y, a synthetic member of the IL-12 cytokine family, suppresses the development of type 1 diabetes in NOD mice. *European Journal of Immunology*.

[46] Özer G., Teker Z., Çetiner S. (2003). Serum IL-1, IL-2, TNF*α* and INF*γ* levels of patients with type 1 diabetes mellitus and their siblings. *Journal of Pediatric Endocrinology and Metabolism*.

[47] Snell-Bergeon J. K., West N. A., Mayer-Davis E. J. (2010). Inflammatory markers are increased in youth with type 1 diabetes: the SEARCH case-control study. *Journal of Clinical Endocrinology and Metabolism*.

[48] Nocoń-Bohusz J., Noczyńska A. (2016). Evaluation the concentration of selected markers of the atherosclerosis process in children with diabetes type 1. *Endokrynologia Pediatryczna*.

[49] Cariello A., Ihnat M., Ross K. (2005). Evidence for a cellular ‘memory’ of hyperglycemic stress. *Diabetes*.

[50] Virk S. A., Donaghue K. C., Cho Y. H. (2016). Association between HbA_1c_ variability and risk of microvascular complications in adolescents with type 1 diabetes. *The Journal of Clinical Endocrinology & Metabolism*.

